# Giant Polypoid Tumor Expressing on the Pyloric Ring

**DOI:** 10.1155/2015/986971

**Published:** 2015-01-22

**Authors:** Hirofumi Sonoda, Takashi Kobayashi, Yuhei Endo, Shoichi Irie, Toru Hirata, Keisuke Minamimura, Ken-ichi Mafune, Masaya Mori

**Affiliations:** ^1^Division of Gastrointestinal Surgery, Mitsui Memorial Hospital, Kanda-Izumi-cho 1, Chiyoda-ku, Tokyo 101-8643, Japan; ^2^Division of Pathology, Mitsui Memorial Hospital, Kanda-Izumi-cho 1, Chiyoda-ku, Tokyo 101-8643, Japan

## Abstract

A 66-year-old Japanese man was referred to our hospital because of suspected duodenal cancer. Upper gastric endoscopy revealed a giant polypoid-type tumor that extended from the duodenum bulb to the pyloric ring. A computed tomography scan revealed a slightly enhanced lobular tumor protruding into the duodenum bulb. Positron emission tomography showed an accumulation of ^18^F-fluorodeoxyglucose in the area extending from the antrum of the stomach to the duodenum bulb. Since an endoscopic ultrasound test suggested that the tumor might invade the muscular tunic, indications of endoscopic mucosal resection were not favored, and the tumor was curatively removed via distal gastrectomy. The histopathologic diagnosis was papillary adenocarcinoma, and the invasion depth was the mucosal layer without vascular invasion, which was different from the preoperative diagnosis. Our case suggests the difficulties in precise diagnosis of the invasion depth of the giant polypoid cancer.

## 1. Introduction

Gastric polypoid tumors vary in terms of histology, neoplastic potential, and management. Preoperative diagnosis is now extremely precise because of technical improvements in upper gastric endoscopy and medical imaging. However, it sometimes differs from the postoperative histopathologic diagnosis. Our case suggests the difficulties in precise diagnosis of the invasion depth of the giant polypoid cancer.

## 2. Case Presentation

**Figure 1:**
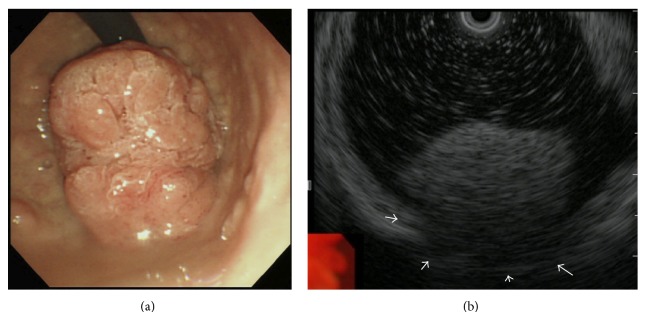
(a) Upper gastric endoscopy revealing a giant polypoid-type tumor extending from the duodenum bulb to the pyloric ring. (b) An endoscopic ultrasound test suggesting that the tumor might invade the muscular tunic (white arrows).

**Figure 2:**
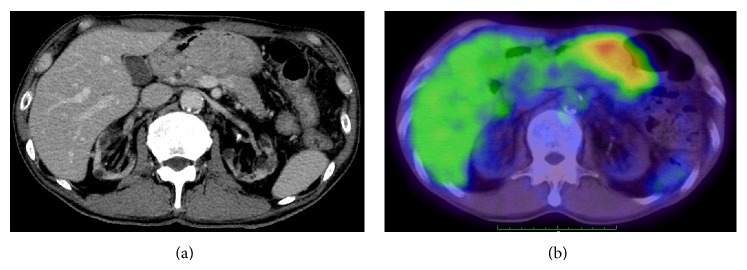
(a) A computed tomography scan revealing a slightly enhanced lobular tumor (34 mm × 54 mm) protruding into the duodenum bulb. (b) Positron emission tomography showing an accumulation of ^18^F-fluorodeoxyglucose (60 mm × 34 mm, maximum standardized uptake value = 5.6).

**Figure 3:**
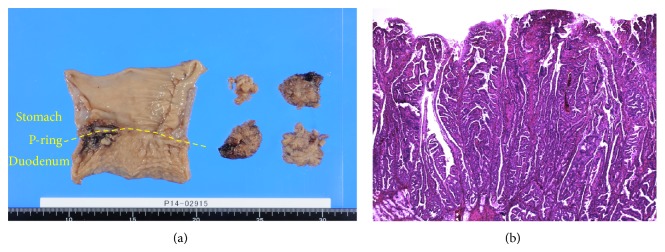
(a) The tumor resected via distal gastrectomy after submucosal resection via an incision in the gastric anterior wall. (b) The histopathologic diagnosis-papillary adenocarcinoma, and the invasion depth is the mucosal layer without vascular invasion.

A 66-year-old Japanese man undergoing hemodialysis for chronic renal failure was referred to our hospital with suspected duodenal cancer. On physical examination, his abdomen was soft and flat without symptoms. Tumor markers were within normal limits, and laboratory findings were normal except for renal dysfunction due to chronic renal failure. Upper gastric endoscopy revealed a giant polypoid-type tumor extending from the duodenum bulb to the pyloric ring; its main location was thought to be the duodenum bulb (Figure 1(a)). The surface of the tumor was rough, but its outline was clear. A biopsy of the tumor showed atypical papillous epithelium containing goblet cells, and nuclei in the epithelium were p53-positive. An endoscopic ultrasound test suggested that the tumor might invade the muscular tunic (Figure 1(b)). A computed tomography scan revealed a slightly enhanced lobular tumor (54 mm × 34 mm) protruding into the duodenum bulb, although serosal invasion was not detected (Figure 2(a)). Positron emission tomography showed an accumulation of ^18^F-fluorodeoxyglucose (60 mm × 34 mm, maximum standardized uptake value = 5.6) in the area extending from the antrum of the stomach to the duodenum bulb (Figure 2(b)). Because indications of endoscopic mucosal resection were not favored, the giant polypoid-type early gastric cancer that was expressed on the pyloric ring and confined within the mucosal layer was curatively removed via abdominal surgery. We performed a submucosal resection via an incision in the gastric anterior wall. The resected tumor was localized in the anterior wall of pyrolic ring. Intraoperative histopathological diagnosis with a frozen section suggested that the tumor contained adenocarcinoma components; therefore, we performed distal gastrectomy followed by Billroth II reconstruction. The extent of resection was from the lesser curvature of stomach, which was 5 cm from the pyrolic ring, to the duodenum, which was 4 cm from the pyrolic ring. The resected lymph nodes were number 3b, number 4d, number 5, and number 6. The histopathological diagnosis was papillary adenocarcinoma, and the invasion depth was the mucosal layer without vascular invasion (Figures 3(a) and 3(b)). There are no metastases in the removed lymph nodes and the numbers of resected lymph nodes were number 3b (0/1), number 4d (0/3), number 5 (0/1), and number 6 (0/4). The pathological stage was T1aN0M0- pStage IA. The postoperative course was uneventful, and the patient was free from recurrence 1 year after the surgery.

## 3. Discussion

Polypoid-type gastric tumors protruding into the duodenum bulb are very rare. Inlaying of the tumor sometimes results in ball-valve syndrome, which is obstruction of the duodenum bulb because of the inlaying of the tumor [1]. In such cases, the reported symptoms are abdominal pain, vomiting, epigastralgia, and a sense of abdominal distension. Gastric polypoid tumors arise from the pyrolic ring or the antrum of the stomach [2] and are sometimes reported as early-stage cancers or leiomyomas. In such cases, they are usually pedunculated, and invasion depth is relatively shallow. In our case, gastrointestinal obstruction did not occur, because the mobility of the tumor was maintained and the tumor was soft. Because the tumor was pedunculated and the invasion depth was the mucosal layer without vascular invasion, it was clearly early-stage.

Gastric polyps are a heterogeneous group of epithelial and subepithelial lesions that vary in terms of histology, neoplastic potential, and management [3]. Treatments for gastric tumors include abdominal surgery, laparoscopic surgery, and endoscopic submucosal dissection, and the choice of operative method depends on the invasion depth of the tumor which is determined via an endoscopic ultrasound test, as well as the neoplastic potential. Recently, it was reported that gastric polypectomy is a safe endoscopic treatment [4]. In our case, the tumor was derived from an adenomatous polyp with high neoplastic potential; therefore, complete polypectomy and sampling of the surrounding mucosa were required. The invasion depth was estimated as the mucosal layer preoperatively; however, endoscopic treatment in our case was technically impossible because the stalk of the tumor was located on the pyloric ring, and working space was limited. Therefore, we firstly chose a submucosal resection via an abdominal incision based on preoperative assessment of a frozen section. However, the definitive diagnosis was adenocarcinoma, so we subsequently performed distal gastrectomy.

The tumor showed an accumulation of ^18^F-fluorodeoxyglucose, and the invasion depth was the muscular tunic as estimated via an endoscopic ultrasound test. However, the histopathologic diagnosis was early cancer of the stomach within the mucosal layer without vascular invasion, which was different from the preoperative diagnosis. This case suggests the difficulties in precise diagnosis of the invasion depth of the giant polypoid cancer.
